# Early-Stage Treatment with Withaferin A Reduces Levels of Misfolded Superoxide Dismutase 1 and Extends Lifespan in a Mouse Model of Amyotrophic Lateral Sclerosis

**DOI:** 10.1007/s13311-014-0311-0

**Published:** 2014-11-18

**Authors:** Priyanka Patel, Jean-Pierre Julien, Jasna Kriz

**Affiliations:** Research Centre of Institut Universitaire en Santé Mentale de Québec, and Department of Psychiatry and Neuroscience, Laval University, 2601 Chemin de la Canardière, Québec, QC G1J 2G3 Canada

**Keywords:** ALS, Neuroinflammation, Withaferin A, SOD1^G93A^, SOD1^G37R^

## Abstract

Approximately 20 % of cases of familial amyotrophic lateral sclerosis (ALS) are caused by mutations in the gene encoding Cu/Zn superoxide dismutase (SOD1). Recent studies have shown that Withaferin A (WA), an inhibitor of nuclear factor-kappa B activity, was efficient in reducing disease phenotype in a TAR DNA binding protein 43 transgenic mouse model of ALS. These findings led us to test WA in mice from 2 transgenic lines expressing different ALS-linked SOD1 mutations, SOD1^G93A^ and SOD1^G37R^. Intraperitoneal administration of WA at a dosage of 4 mg/kg of body weight was initiated from postnatal day 40 until end stage in SOD1^G93A^ mice, and from 9 months until end stage in SOD1^G37R^ mice. The beneficial effects of WA in the SOD1^G93A^ mice model were accompanied by an alleviation of neuroinflammation, a decrease in levels of misfolded SOD1 species in the spinal cord, and a reduction in loss of motor neurons resulting in delayed disease progression and mortality. Interestingly, WA treatment triggered robust induction of heat shock protein 25 (a mouse ortholog of heat shock protein 27), which may explain the reduced level of misfolded SOD1 species in the spinal cord of SOD1^G93A^ mice and the decrease of neuronal injury responses, as revealed by real-time imaging of biophotonic SOD1^G93A^ mice expressing a luciferase transgene under the control of the growth-associated protein 43 promoter. These results suggest that WA may represent a potential lead compound for drug development aiming to treat ALS.

**Electronic supplementary material:**

The online version of this article (doi:10.1007/s13311-014-0311-0) contains supplementary material, which is available to authorized users.

## Introduction

Amyotrophic lateral sclerosis (ALS) is a fatal progressive degenerative disorder characterized by progressive muscle weakness, muscle atrophy, and eventual paralysis, leading to death within 2–5 years. About 5–10 % of patients inherit the disease, typically in an autosomal dominant manner [familial ALS (FALS)]. In 20 % of FALS, missense mutations have been identified in the gene coding for superoxide dismutase 1 (SOD1) [1–3].Various hypotheses have been proposed to explain the toxicity of SOD1 mutants, including protein aggregation [4, 5], oxidative stress [6], mitochondrial dysfunction [7], and excitotoxicity [8]. TAR DNA binding protein 43 (TDP-43) is another protein detected in pathological inclusions of ALS and cases of frontotemporal lobar degeneration with ubiquitin inclusions [9, 10]. Dominant mutations in *TARDBP*, which codes for TDP-43, have been reported by several groups as a primary cause of ALS [11–16], and may account for ~3.0 % of cases of FALS and ~1.5 % of sporadic cases.

Previously, we showed that treatment of a TDP-43 transgenic mouse model of ALS with Withaferin A (WA), an inhibitor of nuclear factor-kappa B (NF-кB) activity, ameliorated disease symptoms and pathological phenotypes such as reduction of denervated neuromuscular junctions and attenuation of neuroinflammation [17]. These findings led us to test WA in mice from 2 transgenic lines expressing different ALS-linked SOD1 mutations, SOD1^G93A^ and SOD1^G37R^. Importantly, recent studies by Frakes et al. [18] have demonstrated that in a SOD1^G93A^ mouse model of ALS, motor neuron death involves activated microglia in a NF-κB dependent manner. WA is a steroid lactone found in the medicinal plant *Withania somnifera*. Semipurified root extract of *W. somnifera* consisting of withanolides and withanosides reversed behavioral deficits, plaque pathology, and accumulation of β-amyloid peptides and oligomers in the brains of amyloid precursor protein/presenilin-1 Alzheimer’s disease transgenic mice [19]. WA exhibits a variety of beneficial effects, including antitumor, anti-inflammatory, and immunomodulatory properties [20]. In addition, WA may act as an inducer of heat shock proteins (Hsps) [21].

Here, we investigated the effects of WA treatment on disease progression and pathological changes in 2 ALS mouse models expressing either SOD1^G93A^ or SOD1^G37R^ mutants. We report that when started early in disease pathogenesis, at time of onset of initial motor function deficits [22, 23], treatment with WA significantly extended the lifespan of SOD1^G93A^ and SOD1^G37R^ mice. WA treatment was associated with a reduction of neuronal stress, attenuated inflammation, upregulation of Hsp25 (mouse ortholog of Hsp27) and Hsp70, and a decrease in levels of misfolded SOD1 species.

## Materials and Methods

### Generation of Glial Fibrillary Acidic Protein–luciferase (luc)/SOD1^G93A^ and Growth-associated Protein-43–luc/Green Fluorescent Protein/SOD1^G93A^ Transgenic Mice

The transgenic glial fibrillary acidic protein (GFAP)–luciferase (luc) mice (FVB/N background) were obtained from Caliper (Caliper Life Sciences, Hopkinton, MA, USA). As previously described [24], the GFAP–luc mice were crossed with the transgenic SOD1^G93A^ transgenic mice (C57/BL6; The Jackson Laboratory, Bar Harbor, ME, USA) to generate double transgenic GFAP–luc/SOD1^G93A^ mice [25, 26]. The genotyping was performed as previously described [27]. The presence of GFAP–luc transgene was assessed by polymerase chain reaction (PCR) with HotStar Taq Master mix Kit (Qiagen, Mississauga, ON, Canada) in 15 mM MgCl_2_ PCR buffer with the following primers: 5′GAAATGTCCGTTCGGTTGGCAGAAGC and 5′CCAAAACCGTGATGGAATGGAACAACA. The presence of the SOD1^G93A^ mutant transgene was assessed by PCR as previously described [27]. To confirm that the transgene copy number of SOD1^G93A^ was not altered in the mice used for this study, the genomic SOD1 levels were evaluated by quantitative reverse transcriptase PCR using genomic DNA isolated from tail tissue. Analysis of the mouse housekeeping gene encoding glyceraldehyde-3-phosphate dehydrogenase was used for normalization purposes. Oligoprimer pairs (used at concentration of 300 nm) were designed by GeneTool 2.0 software (Biotools Inc., Edmonton, AB, Canada) and their specificity was verified by blast in the GenBank database.

The transgenic growth-associated protein (GAP)-43–luc/green fluorescent protein (gfp) reporter mice were generated as described previously [28]. The mice were crossed with the SOD1^G93A^ transgenic mice (C57/BL6; The Jackson Laboratory) to generate double transgenic GAP-43–luc/gfp/SOD1^G93A^ mice [25, 26]. To avoid the effects of genetic background, all experiments were performed on age-matched littermates. Double transgenic mice were genotyped according to the following procedure. The presence of GAP-43–luc/gfp transgene was assessed by PCR of the luciferase reporter gene with the following primers:

- 5′-GGCGCAGTAGGCAAGGTGGT and 5′-CAGCAGGATGCTCTCCAGTTC [29].

All experimental procedures were approved by the animal care ethics committee of Laval University and were in accordance with The Guide to the Care and Use of Experimental Animals of the Canadian Council on Animal Care.

### Analysis of Clinical Symptoms

The onset of weight loss was determined at the time when mice started to exhibit a decline of body weight after reaching a peak. The survival was defined as the loss of righting reflex (the age when the animal could not right itself within 30 s when placed on its side). Measurements of body weight and the loss of hind limb reflex were used to score the clinical onset of disease in SOD1^G93A^ mice, as previously described [30]. The SOD1^G93A^ reflex score and body weight were measured every 2 days, beginning at 45 days. Scoring was performed in a blind manner by animal technicians who had no information about the genotype but had experience in grading SOD1^G93A^ mice paralysis.

### *In Vivo* Bioluminescence Imaging

As previously described, images were gathered using IVIS 200 Imaging System (Xenogen, Alameda, CA, USA) [24, 31]. Twenty minutes prior to the imaging session the mice received an intraperitoneal (i.p.) injection of D-luciferine, a luciferase substrate (150 mg/kg; Xenogen) dissolved in 0.9 % saline. The mice were then anesthetized with 2 % isoflurane in 100 % oxygen at a flow rate of 2 L/min and placed in the heated, light-tight imaging chamber. Images of lumbar spinal cord region of interest were collected using high sensitivity charge-coupled device camera with wavelengths ranging from 300 to 600 nm. Exposition time for imaging was 1 min using different fields of view and a F/1 lens aperture. The bioluminescence emission was normalized and displayed in physical units of surface radiance, photons/s/cm^2^/steradian [31, 32]. The light output was quantified by determining the total number of photons emitted per second using Living Image 4.1 acquisition and imaging software (PerkinElmer, Waltham, MA, USA). Region-of-interest measurements on the images were used to convert surface radiance (photons/s/cm^2^/steradian) to source flux or total flux of photons expressed in photons/s.

### Administration of WA

WA was obtained from Enzo Life sciences (Farmingdale, NY, USA). WA was first dissolved in dimethyl sulfoxide (DMSO) and diluted in 0.9 % saline. The final concentration of DMSO was 10 %. The drug was made fresh every 2 weeks and was protected from light. Male and female transgenic mice and their transgenic littermates were divided randomly into the following 2 groups (*n* =15 per group): 1) transgenic controls, which received vehicle (0.9 % saline with 10 % DMSO); and 2) the transgenic WA treatment group, which received an i.p. injection of WA at a rate of 4 mg/kg body weight, twice a week. The treatment was initiated at early symptomatic stage (40 days of age) as recently proposed by Vinsant et al. [22, 23].

### Tissue Collection and Immunofluorescence Microscopy

Mice were anesthetized by i.p. injection of chloral hydrate (10 mg/ml) and transcardially perfused with 30 ml 0.9 % NaCl, followed by ice-cold phosphate buffered saline (PBS) 1× buffered 4 % paraformaldehyde at pH 7.4. Tissue samples were then postfixed overnight in 4 % paraformaldehyde and equilibrated in phosphate-buffered 20 % sucrose. Spinal cords were cut at a thickness of 25 μm. The double immunofluorescence analysis was performed according to the following procedure. After 1–2 h air drying, sections were blocked in PBS containing 10 % goat serum and 0.25 % Triton X-100 for 30 min. Spinal cord sections were incubated using primary antibodies: 1 : 500 rabbit polyclonal antiglial fibrillary acidic protein (Dako, Carpinteria, CA, USA), 1 : 500 rabbit anti-ionized calcium binding adaptor molecule-1 (Iba-1; Wako Chemicals USA, Richmond, VA, USA), 1 : 50 rabbit polyclonal cyclic adenosine monophosphate-dependent transcription factor (ATF)-3 (Santacruz Biotechnology, Santa Cruz, CA, USA), and 1 : 500 mouse monoclonal neuronal nuclear antigen (Millipore, Temecula, CA, USA). Slides were washed in PBS containing 5 % goat serum and 0.25 % triton X-100, and incubated with the appropriate fluorescent-conjugated secondary antibodies (Alexa; Molecular Probes, Eugene, OR, USA) for 2 h at room temperature. A final wash was performed in PBS and slides were coverslipped with Fluoromount medium (Electron Microscopy Sciences, Hatfield, PA, USA).

### Stereological Counts of Motor Neurons

Sections of horizontal spinal cord were Nissl stained to identify motor neurons in the lumbar spinal cord. The L3–L5 spinal cord sections were individually traced with a 40× microscopic observation and sampled under 400× magnification. The density of labeled cells was estimated by the optical fractionator method using Stereo Investigator software (MBF Biosciences, Williston, ND, USA). The counting parameters were the distance between counting frames (150 μm), the counting frame size (150 μm × 150 μm), the dissector height (10 μm), and the guard zone thickness (1 μm). Motor neurons were identified based on: 1) anatomic location (ventral horn/laminae 9); 2) presence of a distinct nucleolus within the plane of the optical dissector; and 3) a cross-sectional area ≥250 μm^2^. Results are expressed as the total number of motor neuron/mm^3^.

### Immunoprecipitation and Western Blotting

At postnatal day 120, spinal cords were dissected out, rapidly frozen in liquid nitrogen, and stored at –80 °C for cytokine array, immunoprecipitation and Western blot analysis. Whole protein lysates from mouse spinal cords were extracted by homogenization of the tissues in TNG-T lysis buffer (50 mM Tris–HCl pH: 7.4; 100 mM NaCl; 10 % glycerol; 1 % Triton X), sonicated and centrifuged for 20 min at 9000 *g* at 4 °C. Blots were immunostained overnight at 4 °C with primary antibodies, Hsp25/27 (rabbit polyclonal antibody 1 : 2500; Cell Signaling, Danvers, MA, USA), Hsp70, (rabbit polyclonal antibody, clone D69, detects endogenous level of total HSP70 protein, at dilution of 1 : 1000; Cell Signaling), Hsf-1 (rat monoclonal antibody Ab-1, clone 4B4, 1 : 1000; Thermo Scientific, Waltham, MA, USA), Iba-1 (1 : 1000; Wako Chemicals), Toll-like receptor 2 (AB16894, 1 : 1000; Abcam, Cambridge, MA, USA). As previously described [33], immunoprecipitation experiments for misfolded SOD1 were done using the Dynabeads standard protocol (Invitrogen, Carlsbad, CA, USA). Briefly, Dynabeads were washed and coated with the mouse monoclonal antimisfolded SOD1 antibody B8H10 (2 h at room temperature), washed with PBS with Tween 20 and bovine serum albumin/4-(2-hydroxyethyl)-1-piperazineethanesulfonic acid–PBS and incubated overnight with 300 μg spinal cord lysate protein at 4 °C with rotation. After incubation, the beads were washed and fractioned on 14 % sodium dodecyl sulfate polyacrylamide gel electrophoresis (SDS-PAGE).

### Cytokine Array

The expression profile of inflammatory cytokines were performed with a mouse cytokine antibody array (Raybio Mouse Inflammation Antibody Array 1, Cat#AAM- INF-1; RayBiotech, Norcross, GA, USA) as previously described in detail [29]. Protein samples were obtained by homogenization of WA-injected and vehicle-injected SOD1^G93A^ spinal cord (*n* =3) at P120 in 1× cell lysis buffer with protease inhibitor cocktail (#P8340; Sigma, St. Louis, MO, USA) included in the RayBiotech kit. After extraction, samples were spun down at 13,000 rpm for 10 min at 4 °C and supernatant was used for the experiment. For each group (3 mice/group) samples were pooled together and incubated with the array membrane overnight at 4 °C. After washing in the washing buffer (included in the RayBiotech kit), membranes were incubated with biotin-conjugated antibodies overnight. Signal detection was performed according to the RayBiotech protocol, by exposing membranes to x-ray film (Biomax MR1; #8701302; Kodak, Rochester, NY, USA), and the obtained results analyzed using ImageJ software [29]. Data are expressed in arbitrary units relative to appropriate positive control. Statistical analysis was performed by using a 2-tailed unpaired Student’s *t* test.

### Flow Cytometry Analysis

Blood was collected from the submandibular vein of WA- and vehicle-injected mice at 112 and 125 days, as previously described [34], and sent for flow cytometry analyses (Centre hospitalier de l’Université Laval Hospital Research Institute’s Core Flow Cytometry Laboratory). The panel of antibodies (all from BD Biosciences, San Jose, CA, USA) used to evaluate the leukocytes from mice included CD4 (APC Rat Anti-Mouse CD4, clone RM4-5), CD8 (PE-CF594 Rat Anti-Mouse CD8a, Clone 53-6.7), CD25 (FITC Rat Anti-Mouse CD25, clone 7D4), CD45 (V500 Rat Anti-Mouse CD45,clone 3O-f11), FoxP3 (V450 Rat Anti-Mouse FOXP3, clone MF23), interleukin (IL)-4 (PE-Cy 7 rat Anti-Mouse IL-4, clone 11B11), and IL-10 ( PE Rat Anti-Mouse IL-10, clone JEs5-16E3). Samples were analyzed on a flow cytometer (BD LSRII; BD Biosciences) by a blinded individual.

### Statistical Analysis

Data were analyzed using Prism 5.0 (Graph Pad Software, La Jolla, CA, USA). Behavioral data were computed by performing 2-way analyses of variance (except when specified) followed by Bonferroni post-tests and survival data using Mantel–Cox log-rank tests. Optical densities for Iba-1 and GFAP staining, as well as quantification of Western blots, were analyzed by Image J followed by a 2-tailed Student’s *t* test.

## Results

### WA Extends Survival in Transgenic Mice Overexpressing SOD1^G93A^ or SOD1^G37R^ Mutants

**Fig. 1:**
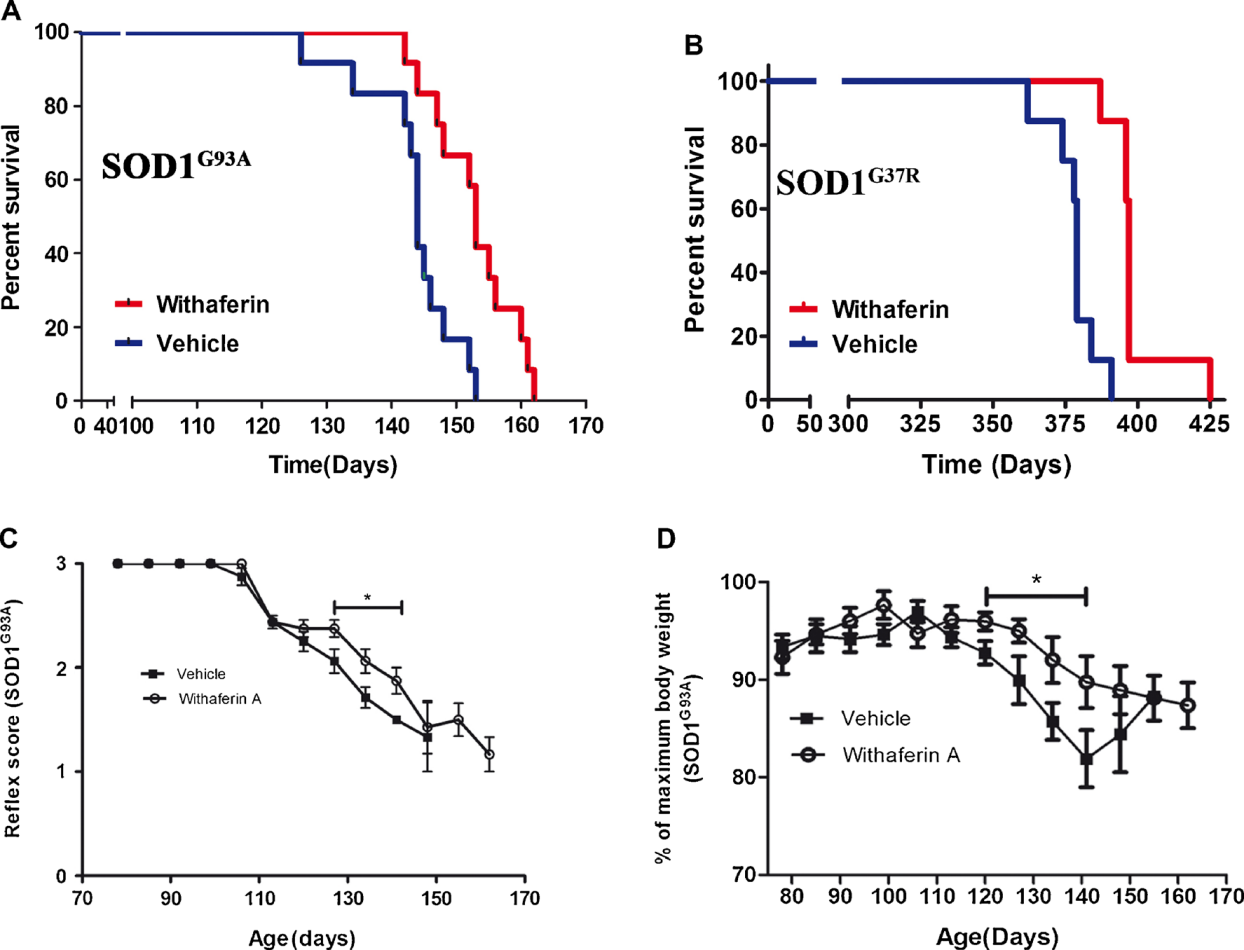
Withaferin A (WA_ extended the survival of superoxide dismutase 1 (SOD1)^G93A^ mice. To examine whether WA can alleviate mutant SOD1-induced neurotoxicity *in vivo*, SOD1^G93A^ and SOD1^G37R^ mice were intraperitoneally injected with WA (4 mg/kg) or vehicle (saline +10 % dimethyl sulfoxide), twice a week from day 40 until the end stage of disease and then statistically analyzed using the Kaplan–Meier method. (A) The Kaplan–Meier survival curve shows that vehicle-treated SOD1^G93A^ (*n* =12) transgenic mice had a mean survival of 144 days, whereas WA-treated mice (*n* =12) lived for 153 days. Log-rank test was statistically significant (*p* <0.01). (B) The Kaplan–Meier survival curve shows that vehicle-treated SOD1^G37R^ (*n* =8) transgenic mice had a mean survival of 379 days, whereas WA-treated mice (*n* =8) lived for 397 days. Log-rank test was statistically significant (*p* <0.01). (C) Hind limb reflex score analysis showed prolonged maintenance of reflex score for particular time points in WA-treated mice. Difference is significant for marked time period. (D) Disease onset was determined by the initial loss of body weight (age of peak body weight). The difference was significant for the marked time period. Each point indicates the mean ± SEM. The data were analyzed by unpaired *t* test

We examined the effect of WA in transgenic mice overexpressing SOD1^G93A^ or SOD1^G37R^ mutants. As described in details in the “Materials and Methods”, starting at postnatal day 40, the SOD1^G93A^ mice were treated with WA twice a week (4 mg/kg i.p.). The mice received continuous treatment until the end stage of disease. A similar therapeutic protocol was applied for the treatment of the SOD1^G37R^ mice. Mice were injected with the same dose, starting at 9 months (early stage of disease) until the end stage of disease. Treatment with WA significantly extended the survival of SOD1^G93A^ mice. Mean survival of vehicle-treated SOD1^G93A^ mice was 145 days (*n* =15), whereas treatment with WA increased the lifespan of SOD1^G93A^ mice to 153 days (*n* =16) (*p* <0.05, a difference of 8 days; Fig. 1A). In the mouse model with slowly progressing disease—the SOD1^G37R^ model—the mean survival of WA-treated SOD1^G37R^ mice was 397 days (*n* =8) compared with controls (379 days; n =8) (*p* <0.01, a difference of 18 days; Fig. 1B). Furthermore, treatment with WA significantly delayed the loss of motor function observed in the motor function tests and prevented the loss of body weight (Fig. 1C, D).

### Reduction of Early Neuronal Injury Response Biophotonic Signals by WA Treatment in GAP-43–luc/gfp/SOD1^G93A^ Mice

Treatment with WA extended survival in 2 different SOD1 mutant mouse models. Therefore, by using a live imaging approach and a cell type-specific reporter mouse, we further investigated potential therapeutic mechanisms and cellular targets. To visualize the effects of WA treatment in SOD1^G93A^ mice in real time we took advantage of the GAP-43–luc/gfp reporter mice, recently generated and validated in our laboratory [28]. Importantly, the results of our recent study revealed that the GAP-43 biophotonic signals imaged from the spinal cords of live SOD1^G93A^ mice may serve as a valid biomarker to assess early neuronal injury response in SOD1 mutant-mediated disease [35]. Moreover, immunofluoresence analysis revealed almost perfect co-relation between GAP-43-driven gfp transgene and ATF-3, known to be upregulated in injured and/or stressed neurons [35–37]. Double transgenic GAP-43–luc/gfp/SOD1^G93A^ mice were generated by crossing heterozygous mice carrying the mutant SOD1^G93A^ transgene with the heterozygous GAP-43–luc/gfp mice co-expressing reporter transgene, luc, and gfp, driven by the murine GAP-43 promoter. In this mouse model, an upregulation of GAP-43 (luciferase expression detectable as a bioluminescence/photon emission and gfp expression detectable by confocal microscopy) can be followed longitudinally in live animals using bioluminescence/biophotonic imaging and a high sensitivity/high resolution charge-coupled device camera.

**Fig. 2:**
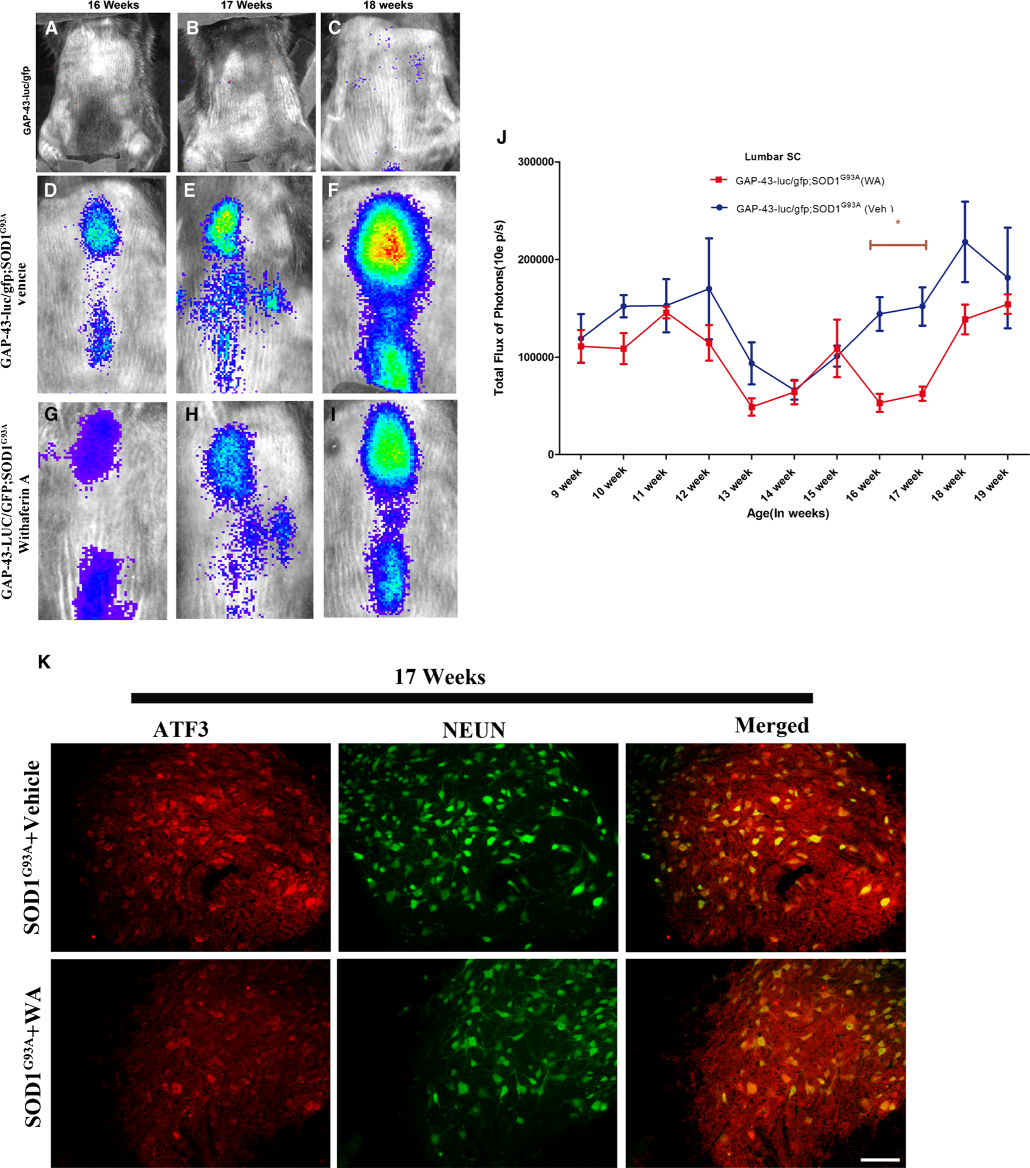
Withaferin A (WA) treatment in growth-associated protein (GAP)-43–luciferase (luc)/green fluorescent protein (gfp)/superoxide dismutase 1 (SOD1)^G93A^ mice reduced neuronal injury biophotonic signals. *In vivo* bioluminescence imaging of GAP-43 induction was analyzed at various time points in spinal cord of GAP-43–luc/gfp/SOD1^G93A^ mice. (A–I) Typical sequence of representative images of spinal cord area obtained from double transgenic mice treated with WA and vehicle (Veh) at different time points (16 and 17 weeks) by *in vivo* imaging (*n* =6 each group) are shown. (J) Longitudinal quantitative analysis of total photon GAP-43 signal/bioluminescence in GAP-43–luc/gfp/SOD1^G93A^ mice in spinal cord. Two-way analysis of variance revealed a statistically significant reduction in bioluminescence signal in the WA-treated group at 16 and 17 weeks (*p* <0.05) We also observed a slight reduction in bioluminescence signal at 18 weeks of age in WA-treated mice. Error bar represents mean ± SEM. (K) Immunofluorescence microscopy using cyclic adenosine monophosphate-dependent transcription factor (ATF3) and neuronal nuclear antigen (NEUN) antibody was performed in spinal cord of WA- and vehicle-treated mice at 17 weeks of age. The ATF3 signal was found to be comparatively less in WA-treated mice than in nontreated mice. Merging of both signals shows co-localization in motor neurons of spinal cord ventral horn. Scale bar 50 μm

To determine the *in vivo* effect of WA treatment on early neuronal injury response, the bioluminescence imaging of the spinal cord was carried out longitudinally on GAP-43–luc/gfp/SOD1^G93A^ double transgenic mice (Fig. 2). WA treatment resulted in significant reduction of the GAP-43 bioluminescence signal in the spinal cord at 16 and 17 weeks of age when compared with vehicle-treated double transgenic mice (Fig. 2D–J). The signal was lower even at week 18 of age (Fig. 2F,I). Reduction of neuronal injury response signal was further confirmed by immunofluorescence microscopy. Analysis of spinal cord sections from WA-treated and control SOD1^G93A^ mice revealed almost perfect co-localization of ATF-3, marker of neuronal injury, and neuronal nuclear antigen staining [38] (Fig. 2K).

### WA Reduced the Level of the Misfolded SOD1 Species and Induced Upregulation of Hsps in SOD1^G93A^ Mice

**Fig. 3:**
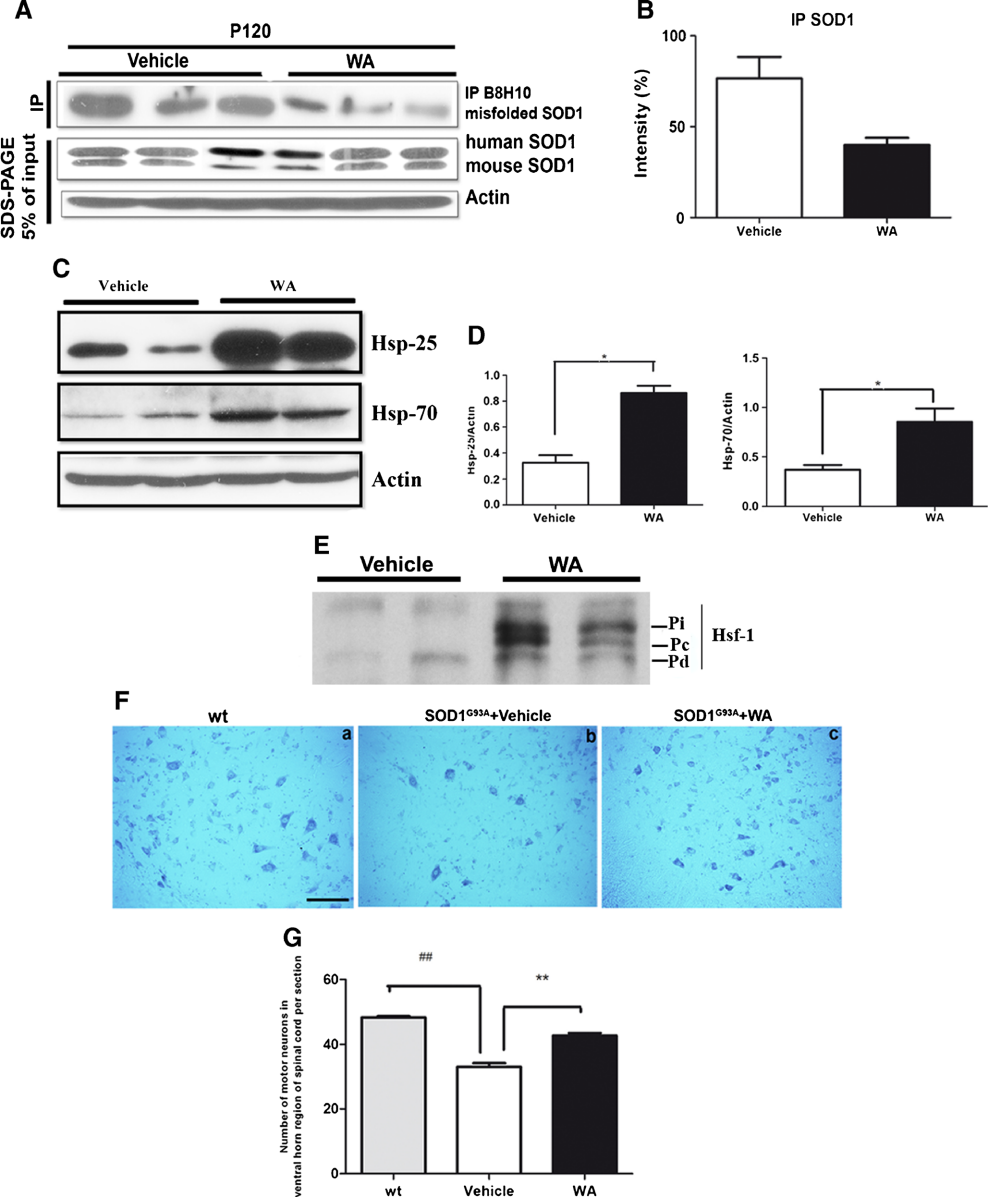
Withaferin A (WA) reduced the level of misfolded superoxide dismutase 1 (SOD1), induced upregulation of heat shock proteins (Hsps), and attenuated loss of motor neurons. (A) Reduced level of misfolded SOD1 in spinal cord of mice. Intraperitoneal (IP) injection of WA led to reduction in the levels of misfolded SOD1 species as detected by immunoprecipitation with B8H10 antibody. Equal amounts of proteins were used as shown on Western blots after sodium dodecyl sulfate polyacrylamide gel electrophoresis (SDS-PAGE) with an actin antibody. Commercial SOD100 polyclonal antibody shows amount of SOD1 protein in all samples. (B) Quantitative densitometric analysis showed a reduced level of misfolded SOD1 protein in WA-treated mice. (C) The protein levels of Hsp25 and Hsp70 in the spinal cord lysates subjected to SDS-PAGE and immunoblotting were compared in the vehicle and WA-injected SOD1^G93A^ mice at postnatal day 120 (P120). Representative immunoblots of Hsp25, Hsp70. (D) Quantitative densitometric analysis of Western blot showed a significant upregulation in the level of Hsp25 in the spinal cord tissue of WA-treated mice (WA: 0.8634 ± 0.05656; vehicle: 0.325 ± 0.05718; *n* =2; *p* <0.01). There was also an increase in the level of Hsp70 protein (WA: 0.854 ± 0.13; vehicle: 0.372 ± 0.45; *n* =2; *p* =0.04). Data represents mean ± SEM. *p* values were derived from Student’s *t* test. All images are from postnatal day 120 mice. (E) Western blot analysis of heat shock transcription factor (Hsf)-1 expression in spinal cord tissue from vehicle-treated SOD1^G93A^ and WA-treated *SOD1* ^G93A^ mice. Normally, the Hsf-1 monomer is present between 65 and 75 kDa, but is activated and shifted by 8–10 kDa in the WA-treated SOD1^G93A^ mice. Pi = inducible phosphorylated Hsf-1; Pc = constitutively phosphorylated Hsf-1; Pd = dephosphorylated Hsf-1. (F) Cross sections of cresyl violet stained hemilumbar spinal cord in wild-type (wt), control SOD1^G93A^, and WA-treated SOD1^G93A^ mice at P120. (G) Quantitative analysis showed that WA-treated SOD1^G93A^ mice contained more motor neurons (42.6 ± 0.8; *n* =3) compared with vehicle-treated SOD1^G93A^ mice (33.00 ± 1.1; *n* =3) (*p* <0.01). Data are mean ± SEM

The misfolded SOD1 species, detectable with specific monoclonal antibodies, have been reported to be among the earliest pathological features in mutant SOD1 mice and are a common hallmark of familial and sporadic ALS [33, 39–43]. Moreover, previous reports suggest that misfolded SOD1 species, detected primarily in affected motor neurons, may serve as a valid biomarker of disease progression [39, 41–43]. The effects of WA treatment on levels of misfolded SOD1 in SOD1^G93A^ mice were examined using a specific antibody (B8H10) against misfolded SOD1 species. Whole protein fractions of the spinal cord lysates prepared from WA-and vehicle-injected SOD1^G93A^ mice at 120 days of age were processed for immunoprecipitation. This was followed by SDS-PAGE and immunoblotting using a polyclonal anti-SOD100 antibody. Remarkably, WA treatment starting at 40 days of age resulted in a 39 % reduction in the levels of misfolded SOD1 in the spinal cord of SOD1^G93A^ mice (Fig. 3A,B). It is noteworthy, that WA, in addition to its anti-inflammatory properties, is known to induce a variety of Hsps [21], and the role of Hsps as intracellular chaperons in protein unfolding/aggregation has been widely established [44]. Hence, we investigated whether the observed decrease in the level of misfolded SOD1 species in WA-treated mice is associated with an increase in the level of Hsps. Although crossing the SOD1^G93A^ mutant mice with Hsp25/27 overexpressors did not significantly affect the course of disease [45], previous work has demonstrated that co-incubation of SOD1^G93A^ with Hsp25/27 can significantly reduce insoluble aggregate formation in cell models of SOD1 aggregation [46]. Moreover, administration of either Hsp25/27 or Hsp70 had neuroprotective effects against SOD1 disease-associated mutant-induced cell death [47]. Therefore, to assess the effects of WA on different elements of cellular stress response, we examined the effects of this compound on the levels of different Hsp family proteins, namely Hsp25 and Hsp70. The levels of Hsp25 and Hsp70 (known to be affected in ALS [48, 49]) were quantified from spinal cord extracts from treated and nontreated SOD1^G93A^ mice at 120 days of age. Western blot analyses revealed a significant, 2.6-fold, upregulation in the levels of Hsp25 and a 2.2-fold upregulation in the level of Hsp70 in SOD1^G93A^ mice treated with WA (Fig. 3C,D). Next, we examined the activation of heat shock transcription factor-1 (Hsf-1) in the spinal cord of WA-treated and untreated mice. Activation of Hsf-1 is characterized by a shift in the Hsf-1 band on Western blot, as Hsf-1 becomes phosphorylated [50]. Western blot analysis of spinal cord lysate from WA-treated SOD1^G93A^ mice showed a shift in the Hsf-1 band that was not observed in vehicle-treated mice (Fig. 3E). Hence, WA induced an increase in survival that was associated with significant reduction in SOD1 misfolded species, marked increase in the levels of Hsp 25 and Hsp70, and, as revealed by *in vivo* imaging, a marked decrease in neuronal early injury response marker GAP-43.

### Neuroprotective Effects of WA in SOD1^G93A^ Mice

Based on our the aforementioned results, we next examined whether WA treatment attenuated the loss of spinal motor neurons in SOD1^G93A^ mice. Cryosections of the lumbar spinal cord (L3–L4) from 120-day-old SOD1^G93A^ mice were Nissl-stained and cells with diameters >25 μm (motor neurons) were quantified [51]. We found 32 % loss of motor neurons in the lumbar spinal cord of SOD1^G93A^ mice when compared with wild-type mice at postnatal day 120 (SOD1^G93A^: 33.0 ± 1.5; wild-type: 48.3 ± 0.3; *p* ≤0.01) (Fig. 3F,G). In contrast, there was only 12 % loss of motor neurons at postnatal day 120 in SOD1^G93A^ mice treated with WA when compared with wild-type mice (42.7 ± 0.9 motor neurons; *n* =3; *p* ≤0.05). Thus, early WA treatment led to a ~30 % increase of motor neuron survival at postnatal day 120 to the end stage of disease (Fig. 3G).

### WA Treatment Suppressed Neuroinflammatory Signals in SOD1^G93A^ Mice

**Fig. 4:**
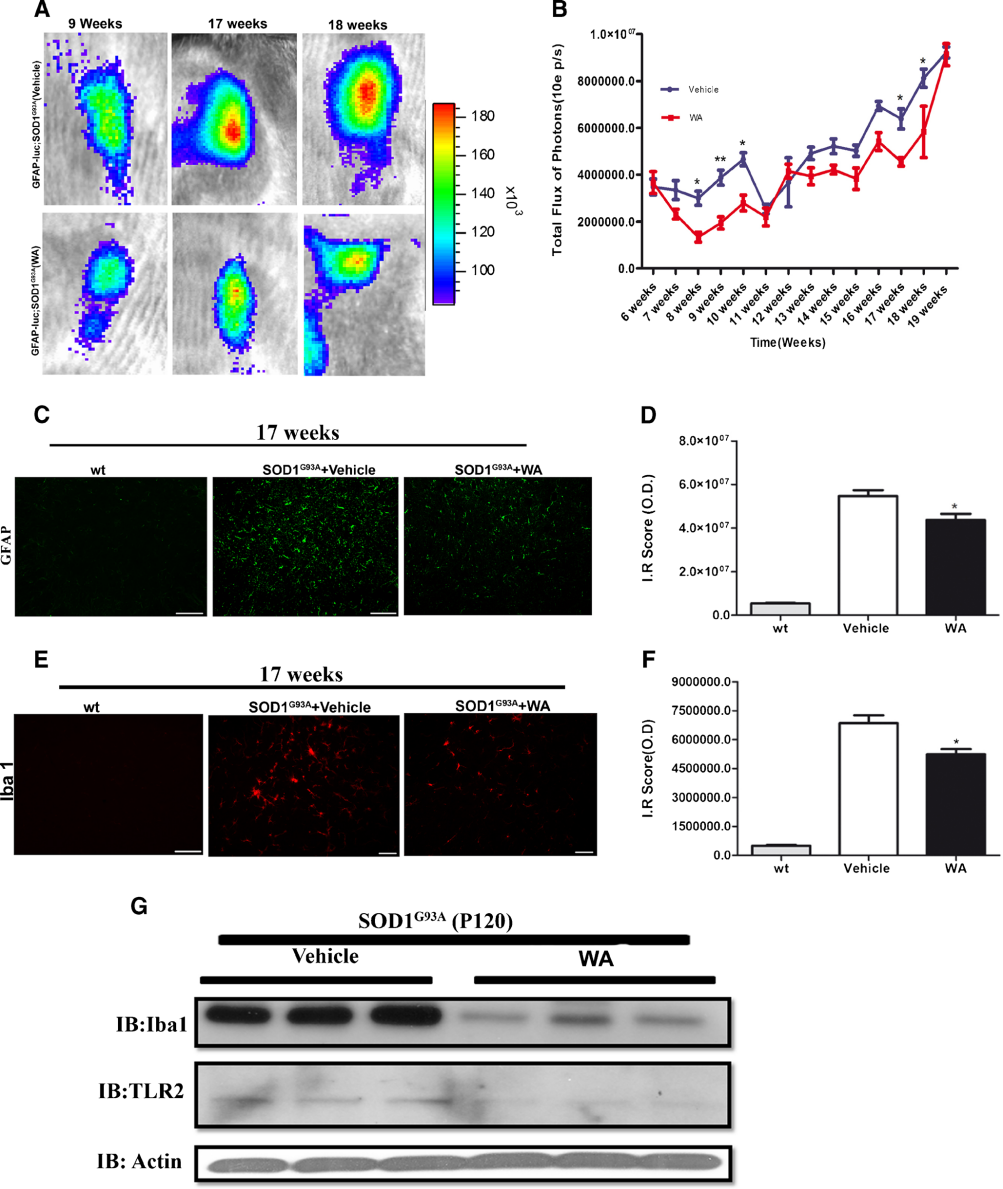
Bioluminescence imaging of astrocyte activation in the spinal cord of glial fibrillary acidic protein (GFAP)–luciferase (luc)/superoxide dismutase 1 (SOD1)^G93A^ mice. (A) Typical sequence of images of spinal cord area obtained from GFAP–luc/SOD1^G93A^ mice at different time points (9, 17, and 18 weeks). (B) Quantitative analysis of the total GFAP signal/bioluminescence (total flux of photon/s) in GFAP–luc/SOD1^G93A^ control mice (blue, *n* =8) and GFAP–luc/SOD1^G93A^ treated (red, *n* =8) at postnatal day 40 revealed that early treatment with WA reduced the GFAP signal at 8, 9, and 10 weeks. A second reduction in GFAP signal after treatment was observed at later stage of disease, at 17 and 18 weeks of age. Two-way analysis of variance revealed a statistically significant reduction in the GFAP signal between the treated and untreated group (*p* <0.05 at 8 and 10 weeks, and, *p* <0.05 at 17 and 18 weeks). Error bar represents mean ± SEM. (C) Photomicrograph of GFAP immunostaining in ventral horn of the spinal cord from wild-type (wt), vehicle, and WA-treated SOD1^G93A^ mice at 120 days (*n* =3 for all groups). (D) Graph represents quantitative analysis of GFAP labeling by measure of optical density (O.D.) (*p* =0.05, *n* =3). (E) Photomicrograph of ionized calcium binding adaptor molecule 1 (Iba1) staining in ventral horn of spinal cord from wt, vehicle, and WA-treated SOD1^G93A^ mice at 120 days. (F) Graph represents quantitative analysis of Iba1 labeling by measure of O.D. (*p* =0.03, *n* =3). (G) Lumber spinal cord lysate from vehicle- and WA-treated SOD1^G93A^ mice at postnatal day 120 (P120) were subjected to immunoblotting (IB) against Iba-1 and Toll-like receptor 2 (TLR2; *n* =3). Actin was used as an internal control (* *p ≤*0.05; ** *p* ≤0.01 by *t* test)

Progressive increase in neuroinflammatory signals is a hallmark of chronic neurodegenerative disorders, including ALS. Namely, the substantial activation of microglial cells and astrocytes is one of the first microscopic findings in the spinal cord sections of patients with ALS and SOD1 mutant mice [52, 53]. Our previous work, using biophotonic/bioluminescence imaging, demonstrated that one of the first signs of disease in SOD1^G93A^ mice is early induction of the biophotonic GFAP signal [24]. Here it is noteworthy that the GFAP gene promoter activity is a target of activated NF-кB and we have previously shown and validated its sensitivity to WA treatments [17]. The *in vivo* effect of WA treatment on astrogliosis was assessed by bioluminescence imaging of luc activity driven by the GFAP promoter in live GFAP–luc/SOD1^G93A^ mice. We injected GFAP–luc/SOD1^G93A^ double transgenic mice with 4 mg/kg body weight of WA twice a week , starting at postnatal day 40 until the end stage of the disease. Analysis of the signal emitted from the spinal cord revealed marked decrease in the luc signal in WA-treated GFAP–luc/SOD1^G93A^ mice at 8–10 weeks compared with nontreated controls (*p* <0.05) (Fig. 4A,B). Another significant decrease in luc signal was observed at 17 and 18 weeks of age (*p* <0.05) in WA-treated mice (Fig. 4B). In line with the obtained *in vivo* imaging results, immunofluorescence analysis of the GFAP staining in spinal cord sections (ventral horn area) from WA-treated SOD1^G93A^ mice at 17 weeks revealed a significant reduction in the signal compared with vehicle-treated SOD1^G93A^ age-matched littermates (*p* <0.05) (Fig. 4C,D). In addition, fluorescence analysis of Iba-1 immunorectivity revealed a significant reduction in spinal cord sections from treated mice compared with the control group, thus suggesting a decrease in microglial activation (*p* <0.05) (Fig. 4E,F). This was further confirmed by Western blot analysis. As shown in Fig. 4G, WA treatment resulted in decreased levels of Iba-1 and Toll-like receptor 2 expression (Fig. 4G). Taken together, our data suggest that WA exerted marked anti-inflammatory effects in the SOD1 mutant model, resulting in decreased astrogliosis and microgliosis.

### WA Alters Cytokine Profiles in Spinal Cord Without Affecting Proliferation and Polarization of Peripheral Immune Cell Population

**Fig. 5:**
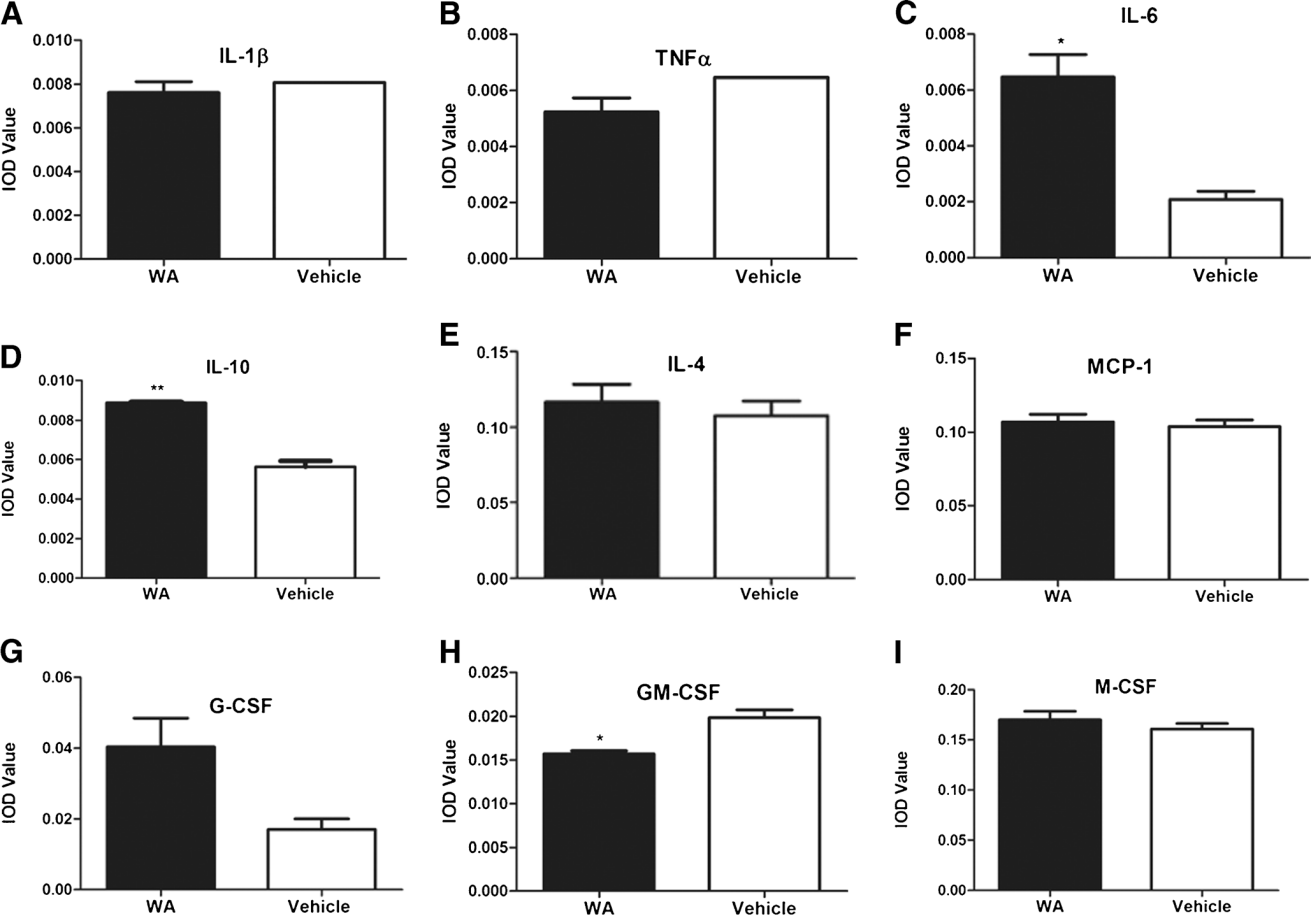
Altered expression profiles of cytokines in spinal cord of superoxide dismutase 1 (SOD1)^G93A^ mice after Withaferin A (WA) treatment. Expression analysis of cytokines as analyzed with cytokine array on protein levels revealed no significant difference between the treated and nontreated group: (A) monocyte chemotactic protein (MCP)-1 (WA: 0.1070 ± 0.005; vehicle: 0.1040 ± 0.004; *n* = ??; *p* =0.70); (B) interleukin (IL)-1β (WA: 0.0076 ± 0.0005; vehicle: 0.0080 ± 0.0000000009; *n* =3; *p* =0.45); (C) tumor necrosis factor (TNF)-α (WA: 0.005 ± 0.0005; vehicle: 0.006 ± 0.000000002; *n* =3; *p* =0.13). However, in the WA-treated group, there was a significant increase in (D) IL-10 (WA: 0.0089 ± 0.0000017; vehicle: 0.0056 ± 0.00029; *n* =3; *p* <0.01); (E) IL-4 (WA: 0.12 ± 0.01; vehicle: 0.1 ± 0.009; *n* =3; *p* =0.61); (F) IL-6 (WA: 0.006 ± 0.0008; vehicle: 0.002 ± 0.0002; *n* =3; *p* =0.04); (G) granulocyte colony stimulating factor (G-CSF) (WA: 0.04 ± 0.008; vehicle: 0.017 ± 0.003; *n* =3; *p* =0.11) ; (H) granulocyte macrophage (GM)-CSF (WA: 0.01 ± 0.0003; vehicle: 0.019 ± 0.00089; *n* =3; *p* =0.05); and (I) macrophage (M)-CSF (WA: 0.1700 ± 0.008481; vehicle: 0.1608 ± 0.005628; *n* =3; *p* =0.46). * *p* ≤0.05; ** *p ≤*0.01 by *t* test. IOD = ??

There is evidence of alterations in expression levels of pro-inflammatory factors such as interferon-γ, tumor necrosis factor (TNF)-α, IL-1β, and granulocyte macrophage (GM) colony-stimulating factor (CSF) in patients with ALS and in mouse models of the disease [54–58]. Previous findings from different experimental paradigms suggest that treatment with WA decreases levels of phospho-p65 and thus attenuates NF-кB-dependent production of proinflammatory cytokines [17, 59, 60]. To examine the effects of WA treatment on the expression profiles of proinflammatory cytokines, we used a standard array of mouse cytokine antibodies to measure over 40 different cytokines from spinal cord extracts of WA- and vehicle-treated SOD1^G93A^ mice at P120 [29]. To our surprise, quantitative analysis revealed no significant changes in the levels of proinflammatory cytokines IL-1β and TNFα (Fig. 5A,B). The WA-treated group exhibited a significant increase in the levels of IL-6 (0.0064 ± 0.0008; *n* =3) compared with those (0.0020 ± 0.0002; *n* =3) in the vehicle-treated controls (Fig. 5C). Interestingly, however, we observed a significant increase in the levels of the key anti-inflammatory cytokine IL-10 (0.0089 ± 0.0000016; *n* =3) compared with vehicle-treated controls (0.0056 ± 0.0003; *n* =3) (Fig. 5D).There was no change in the levels of IL-4 and MCP-1 (Fig. 5E–F,I). No major changes were observed in colony stimulating factors such as granulocyte-CSF and macrophage-CSF, while there was a decreased level of GM-CSF in the WA-treated mice (0.0157 ± 0.0003; *n* =3) when compared with vehicle-treated mice (0.0198 ± 0.0008; *n* =3) (Fig. 5H). Given that WA treatment did not produce marked changes in the proinflammatory cytokine profile, taken together our results suggest that the observed anti-inflammatory effects of WA are owing, instead, to an increase in the levels of the anti-inflammatory cytokine IL-10.

**Fig. 6:**
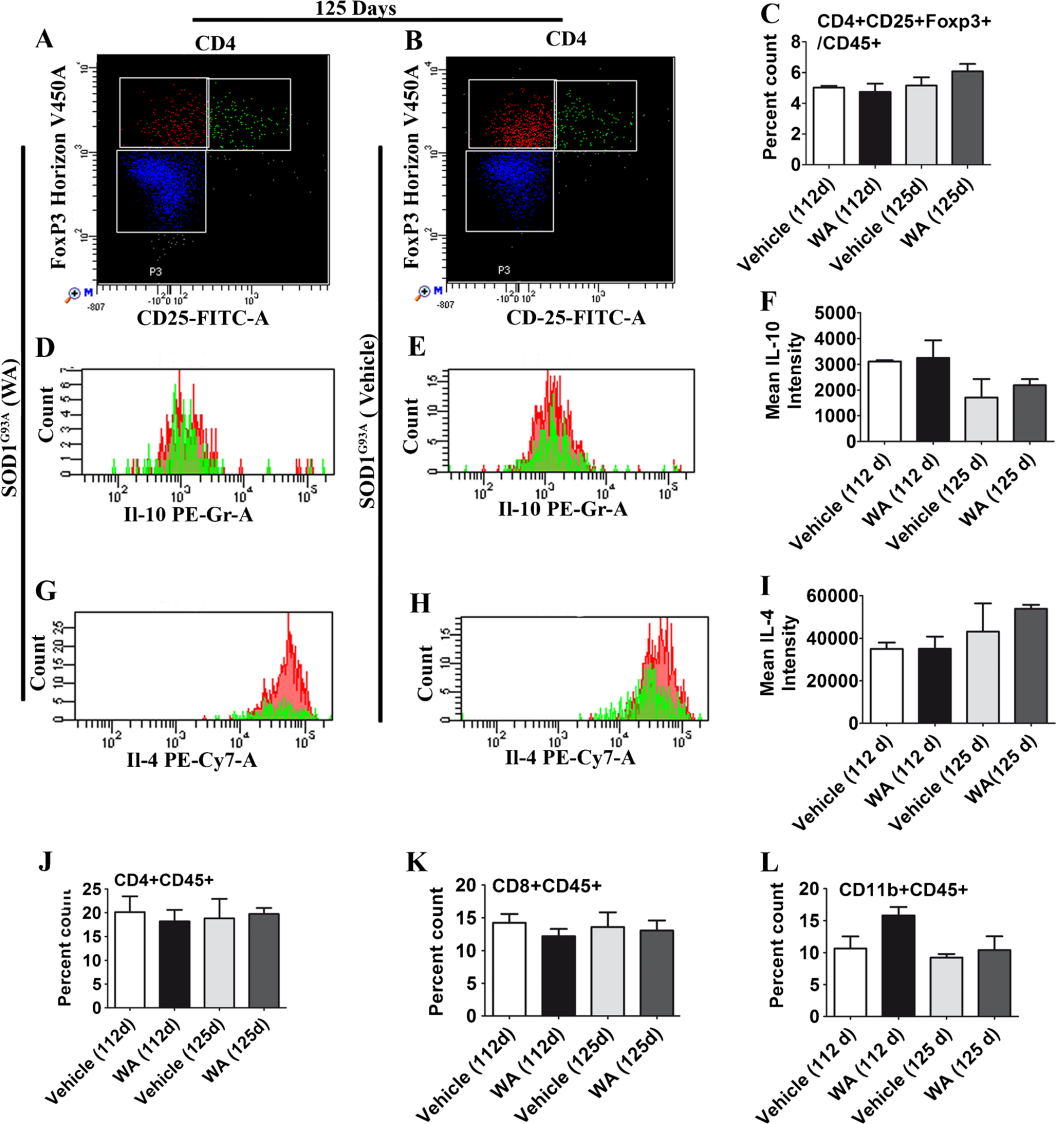
No change in proliferation and polarization of peripheral immune cells in superoxide dismutase 1 (SOD1)^G93A^ mice after Withaferin A treatment. Topographic representation of CD4^+^CD25^+^Foxp3^+^ regulatory T (Treg) cells over CD45^+^ population of (A)WA treated mice and (B) the control group, as analyzed by flow cytometry. (C) Flow cytometry analysis showed no difference in Treg population between the treated and control group at 112 or 125 days. Topographic representation of mean (D, E) interleukin (IL)-10 and (G, H) IL-4 intensity in Tregs of WA-treated and control SOD1^G93A^ mice. Analysis of (F) IL-10 and (I) IL-4 mean intensity in Treg cells in the WA-treated and control groups showed no difference at any mentioned time point. (J, K) No change in the CD4^+^ and CD8^+^ T lymphocyte population at any given time point. (L) Fluorescence-activated cell sorting analysis showed a tendency for a higher CD11b^+^ population in the WA-treated group at 112 days (WA: 15.80 ± 1.30; vehicle: 10.69 ± 1.80; *p* =0.08) but not at 125 days (WA: 10.42 ± 2.10; vehicle: 9.20 ± 0.50; *p* =0.73). FITC = fluorescein isothiocyanate; PE = phycoerythrin

Evidence suggests that WA may affect the ratio and polarization properties of peripheral myeloid cells, including macrophages and T-cells. One of the particular concerns was the potential effects of WA on the subpopulation of regulatory T cells (Tregs) [61]. Namely, it has been well documented that there is an alteration in the population of T lymphocytes (specifically Tregs) in the blood of patients with ALS and in ALS mouse models [62–66]. Tregs are critically involved in suppressing inflammation induced by neurotoxic T lymphocytes and microglia/macrophages, and they play a prominent role in slowing the rate of disease progression in ALS mice [67–70]. Therefore, we analyzed the number of Tregs in the blood by fluorescence-activated cell sorting (FACS) in the WA-treated and nontreated SOD1^G93A^ mice at two time points, postnatal day 112 and postnatal day 125. As Tregs can express anti-inflammatory cytokines, we also measured the levels of IL-4 and IL-10. The Treg transcription factor FoxP3 is currently the most reliable marker for identifying Tregs. Therefore, CD4^+^ CD25^+^FoxP3^+^ Tregs from the WA-treated and control SOD1^G93A^ mice were quantified. As shown in Fig. 6 (A–C) there was no significant difference in the number of Treg cells from the groups of animals at 112 or 125 days. Likewise, there was no difference in the levels of IL-10 (Fig. 6D–F) and IL-4 (Fig. 6G–I) between WA-treated and control group. In addition, we also investigated for possible shift in CD4^+^ and CD8^+^, as well as the CD11b^+^, population. The quantitative FACS analysis revealed no changes in the CD4^+^ and CD8^+^ T lymphocyte population at any mentioned time point (Fig. 6J,K). At the initial time point of 112 days we observed a slight and transient increase in the CD11b^+^ population. However, the observed changes did not reach statistical significance (Fig. 6L).

### No Beneficial Outcome with Late Initiation of WA Treatment

**Fig. 7:**
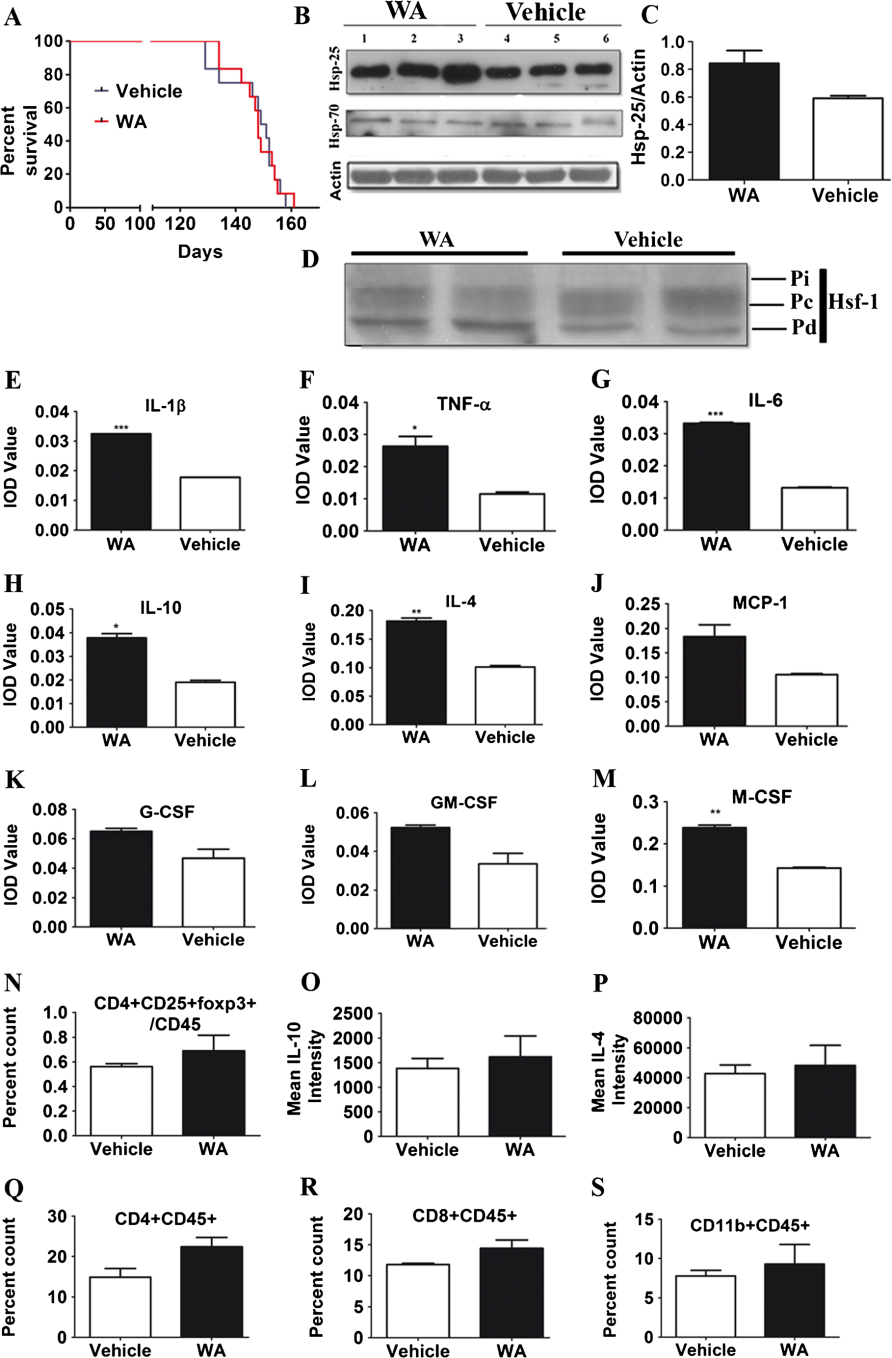
Withaferin A (WA) treatment at late stage of disease did not affect the course of disease and/or survival of superoxide dismutase 1 (SOD1)^G93A^ mice. (A) The graph represents the probability of survival of untreated SOD1^G93A^ mice (*n* =12) and WA-treated mice with onset at 90 days (13 weeks; *n* =12). Median survival times were not significantly different (150 *vs* 148 days, respectively; *p* =0.98). (B) The protein levels of heat shock protein (Hsp)25 in the spinal cord lysates subjected to sodium dodecyl sulfate polyacrylamide gel electrophoresis and immunoblotting were compared between the WA- and vehicle-injected SOD1^G93A^ mice on postnatal day 120 (P120). Representative immunoblots for Hsp25 and Hsp70 and actin as loading control are shown. (C) Quantitative densitometric analysis of Western blots did not show a significant upregulation in the level of either Hsp25 or Hsp70 in the WA-treated mice (*p* >0.05). (D) Representative Western blot of heat shock transcription factor (Hsf)-1 expression in spinal cord tissue from WA-treated and untreated SOD1^G93A^ mice. Pi = induced phosphorylated Hsf-1; Pc = constitutively phosphorylated Hsf-1; Pd = dephosphorylated Hsf-1. (E–M) Expression analysis of different cytokines (protein level) between WA- and vehicle-treated SOD1^G93A^ mice. Levels of (E) interleukin (IL)-1β, (F) tumor necrosis factor (TNF)-α, and (G) IL-6 were significantly increased after WA treatment (IL-1β—WA: 0.03 ± 0.00000015; vehicle: 0.017 ± 0.00000003; TNF-α—WA: 0.026 ± 0.003; vehicle: 0.011 ± 0.0006; IL-6—WA: 0.033 ± 0.0002; vehicle: 0.013 ± 0.0002). Levels of (H) IL-10 and (I) IL-4 were also increased in WA-treated animals (IL-10—WA: 0.037 ± 0.0018; vehicle: 0.019 ± 0.0008; IL-4—WA: 0.18 ± 0.005; vehicle: 0.1 ± 0.0026) There was no change in levels of (J) monocyte chemotactic protein (MCP-1), (K) granulocyte colony stimulating factor (G-CSF), (L) and granulocyte macrophage CSF (GM-CSF), while levels of (M) macrophage CSF (M-CSF) were significantly increased in the WA-treated group (WA: 0.23 ± 0.006; vehicle: 0.14 ± 0.0017). Fluorescence-activated cell sorting (FACS) analysis showed no change in the population of (N) regulatory T cells, or mean intensity of (O) IL-10 or (P) IL-4 in the blood lymphocytic population between WA- and vehicle-treated groups. Analysis of (Q) CD4^+^ and (R) CD8^+^ lymphocytes, and (S) the CD11b^+^ population in blood by FACS showed no significant change. * ??; ** ??; *** ??

We next investigated whether initiation of WA treatment at a later stage of disease would also provide neuroprotection and extend survival in SOD1 mutant mice. We carried out the same injection protocol of WA in SOD1^G93A^ mice with initiation of treatment at 90 days of age. Our results revealed no significant difference in the mean survival time between the vehicle-treated (150 days; *n* =12) and WA-treated group of SOD1^G93A^ mice (148 days; *n* =12) (*p* =0.97) (Fig. 7A). Because neuroprotection with early treatment with WA correlated with an increase in Hsp25 and Hsp70 levels, we next asked whether similar protective mechanisms are induced in the later-stage initiation protocol. Remarkably, no significant changes and marked upregulation of Hsp25 or Hsp70 levels in the spinal cord lysates occurred when WA treatment was initiated at 90 days of age (Fig. 7B,C). As shown in Fig. 7D, this poor Hsp response was clearly associated with the lack of activation of transcription factor Hsf-1, as revealed by lack of gel shift/Hsf-1 phosphorylation in spinal cord samples.

Finally, to assess the effects of late treatment initiation (postnatal day 90) with WA on cytokines profiles, we evaluated the levels of different pro- and anti-inflammatory cytokines in the spinal cord of WA- and vehicle-treated mice at postnatal day 120. Our results confirmed an altered cytokine profile but, surprisingly, we observed alterations in levels of both anti- and proinflammatory groups of cytokines. Quantitative analysis revealed major changes in the levels of proinflammatory cytokines such as IL-1β, TNF-α, and IL-6, between the 2 experimental groups (Fig. 7E–G). Remarkably, quantitative analysis showed a significant increase in the levels of anti-inflammatory cytokines in WA-treated group (Fig. 7H–J). In addition, levels of macrophage-CSF was also significantly increased in the WA treatment group with no significant change in levels of GM-CSF and granulocyte-CSF (Fig. 7K–M).

We next analysed the effect of late WA treatment on the population of T lymphocytes in the blood of SOD1^G93A^ mice by flow cytometry. There was no significant difference in the population of Tregs, or IL-10 and IL-4 levels in blood between WA-treated and control animals (Fig. 7N–P), and quantitative analysis revealed no significant changes in the populations of CD4^+^, CD8^+^, and CD11b ^+^ at the periphery (Fig. 7Q–S). Taken together, our results suggest that when administered at advanced disease stage, WA was unable to induce significantly Hsp25 and Hsp70. Surprisingly, late initiation of WA treatment increased both anti- and proinflammatory cytokine levels in the spinal cord tissue. The late WA treatment did not have significant impact on the peripheral immune cells/immune response.

## Discussion

Our previous study revealed the beneficial effects of WA, including a reduction of inflammation and amelioration of motor deficits in a mouse model of ALS based on overexpression of the human TDP-43 transgene [17]. Here, we report that WA treatment conferred neuroprotective effects with extension of lifespan in 2 mouse models of ALS with overexpression of different mutant SOD1 (SOD1^G93A^ or SOD1^G37R^) (Fig. 1). WA was effective only when treatment was initiated early in disease pathogenesis, at the time of onset of motor function deficits, as recently reported by Vinsant et al. [22, 23].

Our analyses of SOD1^G93A^ mice suggest that WA may exert protective effects through multiple pathways. It is well established that WA exerts potent anti-inflammatory effects [71–73], and our results confirmed that WA can reduce neuroinflammation in SOD1^G93A^ mice when treatment is initiated at early stage of disease. For instance, we took advantage of double transgenic GFAP–luc/SOD1^G93A^ mice in which astrocyte activation can be visualized throughout disease progression [24]. The results of our *in vivo* imaging revealed an attenuation of astrogliosis by WA treatment at 8–10 weeks of age and then at 17 and 18 weeks of age in SOD1^G93A^ mice (Fig. 4A,B). Immunofluorescence microscopy and immunoblotting further confirmed a decrease in GFAP and of Iba-1 signals in 17-week-old SOD1^G93A^ mice treated with WA (Fig. 4C,D). As activated astrocytes and microglia can produce a variety of cytokines, with some having harmful effects [56], we further determined the effect of WA treatment on cytokine expression pattern in the spinal cord of SOD1^G93A^ mice. Interestingly, early WA treatment resulted in a significant increase in the levels of IL-10 in lumbar spinal cord of SOD1^G93A^ mice at 120 days of age (Fig. 5D). IL-10 is known to confer beneficial effects in several neuroinflammatory disease models, including experimental autoimmune encephalomyelitis, traumatic or excitotoxic spinal cord injuries, stroke, and Parkinson’s disease [74–78]. Conversely, WA caused a downregulation in the level of GM-CSF in spinal cord of SOD1^G93A^ mice (Fig. 5H). GM-CSF is a proinflammatory cytokine, upregulated in various neurological disorders, such as Alzheimer’s disease, vascular dementia, and multiple sclerosis [79–81]. Thus, the reduction of inflammation by WA treatment in SOD1^G93A^ mice may be owing, in part, to an upregulation of anti-inflammatory cytokine IL-10 and by a downregulation of proinflammatory cytokine GM-CSF. Moreover, we carried out FACS analysis of the blood to examine the effect of WA treatment on the lymphocyte population, specifically Tregs. A previous study on SOD1^G93A^ mice revealed that the numbers of Treg cells are increased at early slowly progressing stages, augmenting IL-4 expression, and are then decreased when the disease rapidly accelerates, possibly through the loss of FoxP3 expression [67]. In patients with ALS, the numbers of Tregs and expression levels for FOX-3 and IL-4 were inversely correlated with disease progression rates [82]. However, our FACS analysis revealed no effect of WA on the number of Tregs in the blood or on thee levels of IL-10 or IL-4 (Fig. 6F–I). Taken together, our data provided no evidence of protective inflammatory responses through a modulation of peripheral Tregs.

The levels of misfolded SOD1 species in the spinal cord have been used as a valuable indicator of disease progression [40]. Immunotherapeutic approaches aiming to reduce the levels of misfolded SOD1 species have been effective in delaying disease onset and progression in SOD1^G93A^ mice [33, 35]. An upregulation of Hsps with the ensuing reduction in levels of misfolded SOD1 may constitute another mechanism by which WA may confer neuroprotection in SOD1^G93A^ mice. As shown in Fig. 3 (C,D), WA treatment significantly increased the amount of Hsp25 (a mouse ortholog of Hsp27) in the spinal cord of SOD1^G93A^ mice, which is line with a report that WA is an inducer of Hsps. Many reports have shown that Hsp27/25 protects against neuronal damage induced by FALS-related SOD1 mutant [46, 47, 83–86]. Moreover, Hsp27/25 was found to inhibit the *in vitro* aggregation of SOD1^G93A^ proteins [46]. Thus, an upregulation of Hsp25 in WA-treated SOD1^G93A^ mice may explain, in part, the reduction in levels of misfolded SOD1 species as determined by immunoprecipitation with the specific B8H10 antibody (Fig. 3A,B) and increased number of surviving motor neurons (Fig. 3F,G).

The combined results revealed an effective therapeutic effect of WA when treatment is initiated at onset of motor deficits in SOD1^G93A^ mice, which has recently been reassessed to be at 30–40 days of age according to leaded grid test and treadmill gait analysis [22, 23]. However, when WA treatment was initiated at a later stage of disease (90 days of age), at a time coincident with detection of motor neuron death [22, 23], there was no beneficial effect on the survival of SOD1^G93A^ mice (Fig. 7A). As shown in Fig. 7B, when administered after disease onset, WA lost its ability to upregulate Hsp25 and Hsp70. Interestingly, previous work by Maatkamp et al. [48] revealed that in SOD1^G93A^ mutant mice, a decrease in Hsp25 protein expression precedes degeneration of large motor neurons. Taken together, these data suggest that a therapeutic intervention for ALS based on WA medication (and possibly some other therapeutic approaches) would need to be initiated early in the pathogenic process at time when cellular responses to stress or to inflammatory signals are still adequate. For instance, a late-onset initiation of WA administration in SOD1^G93A^ mice caused increases in both anti- and proinflammatory cytokines (Fig. 7E–M), suggesting a marked deregulation of immune system responses at a late stage of disease.

If started at early disease stage, WA should be effective in attenuating deleterious neuroinflammatory responses and in conferring neuroprotection partly through an upregulation of Hsp25 and reduction of misfolded protein species. WA is a steroid lactone present in a medicinal plant, *W. somnifera*, which has been used for centuries in Ayurvedic medicine. The therapeutic effects of WA in various ALS mouse models suggest that WA should be considered as a promising lead compound for drug development aiming to treat ALS.

## Electronic supplementary material

Below is the link to the electronic supplementary material.

ESM 1 (PDF 1224 kb)
